# Commentary: Shaping men's memory: the effects of a female's waist-to-hip ratio on men's memory for her appearance and biographical information

**DOI:** 10.3389/fpsyg.2017.00157

**Published:** 2017-02-08

**Authors:** David S. Smith

**Affiliations:** Department of Psychology, School of Health, BPP UniversityLondon, UK

**Keywords:** episodic memory, adaptive memory, mate selection, selection pressures, gender differences

“But what the hell is it for?” asked Alan Baddeley (1988) about human memory. From an adaptive perspective, our mnemonic systems ought to be calibrated to retain information of high adaptive value i.e., that which promotes individual survival and reproduction (Tooby and Cosmides, 1989; Klein et al., 2002). To address the former, the last decade has seen the emergence of an extensive functionalist literature providing evidence of a survival mode in episodic memory (for a review, see Nairne and Pandeirada, 2016). These experiments characterize memory as the process of a general survival optimization system, attuned toward relevant stimulus.

Regarding the latter part of nature's criterion, labs have examined the role of memory in courtship by exploring how contextual factors including jealousy (Maner et al., 2009), relationship status (Karremans et al., 2011), and primed infidelity (Schutzwohl and Koch, 2004) influence recall. Furthermore, generalized memory enhancements have been observed in response to attractive female faces (Maner et al., 2003; Becker et al., 2005). Inter-sex biases in men were interpreted as reflecting sensitivity to potential mates, while intra-sex biases in women were interpreted as serving to catalog quality competitors. Although these studies point toward functionally specialized cognitive modules, Fitzgerald et al. (2016) argue a focus on facial attractiveness is limited, since it is not the sole arbiter of mating decisions. Other potentially useful features include clothing, body shape, and social information (Buss, 1989). Moreover, physical characteristics of potential mates are only one component of the representation that is formed during an encounter. In addition to the appearance of who was there, people will need to remember that something occurred (what) at a particular time (when) in a particular place (where) (Nairne, 2015).

Before Fitzgerald et al. (2016) few papers showed a correspondence between mate-choice relevant cues and memory enhancements for related stimulus (for exceptions, see Allan et al., 2012; Smith et al., 2012, 2013). Those that had done so exclusively identified effects in women, even in instances where data was gathered for men. Women's recall was consistently better for incidental details associated with men boasting features congruent to their mating strategies vs. less desirable competitors, i.e., masculine features for short-term preferences, and feminine features for long term (Gangestad and Simpson, 2000; Fink and Penton-Voak, 2002). In these studies, it was hypothesized that the greater cost of unsuccessful courtship facing women would have necessitated the need to more easily retrieve memories, from a variety of past contexts, into the personality and behavior of men.

To my knowledge, Fitzgerald et al. (2016) were first to discover biases in men's memory, for concurrent information, prompted by cues of mate quality in women. Crucially the task demands diverged from previous paradigms. Across two studies they showed men variants on the same image of a woman in black, with an accompanying paragraph of biographical information. This included her name, college major, job, and places she would like to visit, among other things. Next, participants' memory for details about the target and their appearance was assessed as a function of her waist to hip ratios (WHR). This cue was selected (a) to counter the limited focus on facial/vocal displays and (b) its apparent role in health, fecundity, and cognitive ability (Singh, 1993). Digital manipulations were used to give her a measurement between 0.50 and 0.90. In both free recall and recognition conditions, participants who viewed the model with a WHR of 0.50 or 0.90 recalled and recognized fewer personal details than those who saw her with a WHR of 0.60, 0.70, or 0.80. Memory data was complemented by preference data in which women with a WHR of 0.70 received the highest ratings. Elsewhere men have identified this measurement as particularly attractive (Singh, 2002; Dixson et al., 2011).

The authors theorize that their data differs from previous findings because they tested memory for information explicitly linked to a target, instead of it being incidental. Parental investment theory (Trivers, 1972) stipulates that asymmetric courtship costs mean men's optimal mating strategies emphasize quantity vs. quality (Kenrick et al., 1990; Buss and Schmitt, 1993). That is to say, men are expected to devote a larger proportion of their mating effort toward short vs. long term relationships. Thus, Fitzgerald et al. (2016) anticipate that a functional memory system in men should be oriented toward information useful for determining a prospect's mating value. Cited candidates include physical features, biographical data, values, and demographic background. A memory system dedicated toward retaining these features may heighten fitness-relevant decisions by helping men to identify suitable partners.

In contrast, Smith et al. (2012) propose a functional system for women should aid comparative vs. absolute evaluation (Bateson and Healy, 2005). It should therefore be geared toward indexing men's behavior over time instead of first impressions. Female participants' memory biases were for subsidiary details, implying a generalized effect following increased arousal/attention, during episodes when a preferred male was present. This information potentially helps them assess mates against (a) assumptions of their behavior, as signaled morphologically (Perrett et al., 1998), and (b) absent competitors. In instances when men defy expectations, women potentially forfeit good genes, or get misled by a mate signaling an investment they do not later meet. Both outcomes are maladaptive. Hence memory should prioritize what desirable men say or do vs. who they are *per se* (Allan et al., 2012; Smith et al., 2013). Data from Horgan et al. (2015) suggests this pattern is true to the extent that women are long term oriented.

Research into sex-specific memory biases for mate-choice relevant stimulus is still in its infancy. Yet it is encouraging to see evidence for the impact of delineated selection pressures between men and women. The data from Fitzgerald et al. (2016) is significant because it addresses a discrepancy in the adaptive memory literature. It further extends social perception research, surrounding men's mating strategies, into the cognitive domain, building on the functionalist framework proposing a sociosexual role of memory. In doing so the authors also bring us closer to answering Baddeley's question.

## Author contributions

The author confirms being the sole contributor of this work and approved it for publication.

### Conflict of interest statement

The author declares that the research was conducted in the absence of any commercial or financial relationships that could be construed as a potential conflict of interest.
